# DNA methylation and immune evasion in triple-negative breast cancer: challenges and therapeutic opportunities

**DOI:** 10.3389/fonc.2025.1534055

**Published:** 2025-02-06

**Authors:** Wen-yu Cai, Xin-xian Cai, Yi-ran Fei, Rui Ye, Ding-ming Song, Dan Hu, Wan-wan Zhang, Ming-fei Xia, Xiao-xiao Yang

**Affiliations:** ^1^ The First Affiliated Hospital of Zhejiang Chinese Medical University (Zhejiang Provincial Hospital of Chinese Medicine), Hangzhou, China; ^2^ School of Medical Technology and Information Engineering, Zhejiang Chinese Medical University, Hangzhou, China; ^3^ The First Clinical Medical College, Zhejiang Chinese Medical University, Hangzhou, China; ^4^ Department of Urology, Jinzhou Medical University, The First Hospital of Jinzhou Medical University, Jinzhou, Liaoning, China; ^5^ Department of Clinical Lab, The Cixi Integrated Traditional Chinese and Western Medicine Medical and Health Group Cixi Red Cross Hospital, Cixi, China

**Keywords:** triple-negative breast cancer, DNA methylation inhibitors, immune checkpoint inhibitors, immune evasion, epigenetic therapy, personalized medicine

## Abstract

Triple-negative breast cancer (TNBC) is an aggressive subtype of breast cancer characterized by the lack of estrogen receptor (ER), progesterone receptor (PR), and human epidermal growth factor receptor 2 (HER2). Chemotherapy remains the primary treatment option, yet TNBC frequently develops resistance, leading to relapse and metastasis. Emerging evidence highlights the potential of combining DNA methylation inhibitors with immune checkpoint inhibitors (ICIs). DNA methylation contributes to immune escape by silencing immune-regulatory genes, thereby reducing the tumor’s visibility to immune cells. Reversing this epigenetic modification can reinvigorate immune surveillance and enhance the efficacy of immunotherapies. This review discusses the role of DNA methylation in TNBC progression and immune evasion, focusing on recent advances in combination therapies involving DNA methylation inhibitors and ICIs. We discuss the underlying mechanisms that enable these therapeutic synergies, preclinical and clinical evidence supporting the approach, and the challenges posed by tumor heterogeneity, drug resistance, and toxicity. Finally, we explore the potential for personalized treatment strategies incorporating multi-omics data to optimize therapeutic outcomes. The integration of epigenetic therapies and immunotherapy offers a promising avenue for improving survival in TNBC patients.

## Introduction

Breast cancer is a heterogeneous disease comprising several subtypes, with Triple-Negative Breast Cancer (TNBC) representing approximately 10-15% of all cases (1). Unlike other subtypes, TNBC lacks expression of ER, PR, and HER2 (2). The absence of these critical receptors makes TNBC non-responsive to hormone-based therapies and correlates with higher metastatic potential and shorter overall survival rates (3–6). TNBC tumors are often more prone to early metastasis, particularly to visceral organs, and show poor differentiation at the cellular level. The tumor microenvironment, including immune cells and stromal interactions, is crucial in driving TNBC’s aggressiveness (7). TNBC is also associated with high genetic and phenotypic heterogeneity, complicating treatment efforts (8).

TNBC comprises multiple subtypes, including basal-like 1 (BL1), basal-like 2 (BL2), mesenchymal (M), mesenchymal stem-like (MSL), and luminal androgen receptor (LAR) subtypes. The BL2 subtype, enriched in growth factor signaling pathways, often exhibits stronger resistance to chemotherapy, whereas the BL1 subtype typically shows greater sensitivity to DNA-damaging agents (9). The LAR subtype is characterized by low immune cell infiltration and M2 macrophage activity, which are associated with poorer responses to immunotherapy and worse prognoses (10). In contrast, the BL1 and BL2 subtypes may benefit more from immunotherapy due to higher immune activation markers.

Chemotherapy remains the mainstay treatment for TNBC, but it is fraught with challenges. Despite being more sensitive to initial chemotherapy compared to other breast cancer subtypes, TNBC frequently develops resistance, leading to relapse and metastasis (8). Various molecular pathways often mediate this resistance, such as the overexpression of ATP-binding cassette (ABC) transporter proteins, which actively efflux chemotherapy drugs from cancer cells (11). The current standard of care for TNBC involves anthracycline and taxane-based chemotherapy regimens. While these therapies have demonstrated efficacy in early-stage disease, their effectiveness diminishes significantly in relapsed and metastatic cases due to the emergence of multi-drug resistance (2). Moreover, newer agents like immune checkpoint and PARP inhibitors have shown promise, but only a subset of patients respond favorably, leaving a substantial proportion with limited options (12). As a result, ongoing research is focused on identifying novel targets and combination therapies to overcome resistance and improve outcomes in TNBC patients.

## Emergence of immunotherapy in cancer treatment

Cancer immunotherapy has rapidly expanded, revolutionizing treatment strategies across multiple malignancies. The main targets of these therapies are inhibitory receptors such as programmed death-1 (PD-1), programmed death-ligand 1 (PD-L1), and cytotoxic T-lymphocyte-associated protein 4 (CTLA-4) (13–15). These molecules play a crucial role in downregulating immune responses, which tumors exploit to escape immune surveillance. Blocking these pathways has significantly improved outcomes for various cancers, including melanoma, non-small cell lung cancer, and renal cell carcinoma (16).

The first immune checkpoint inhibitors were approved for advanced-stage melanoma, showing long-lasting remissions in previously untreatable patients (17). PD-1 inhibitors such as nivolumab and pembrolizumab, as well as CTLA-4 inhibitor ipilimumab, have demonstrated durable responses across multiple cancer types by releasing the “brakes” on the immune system, allowing T cells to attack cancer cells more effectively. However, these treatments are not without challenges, as only a subset of patients exhibit favorable responses due to factors such as tumor mutation burden and the immunosuppressive tumor microenvironment (18).

## Limited efficacy of immunotherapy as a monotherapy in TNBC

Despite the success of immune checkpoint inhibitors in other cancer types, their efficacy as monotherapy in TNBC has been limited. Although the introduction of ICIs targeting PD-1 and PD-L1 has shown promise, especially in combination with chemotherapy, the immune landscape of TNBC often presents substantial barriers to effective treatment (19, 20).

One major limitation is immune evasion. These include the recruitment of immunosuppressive cells such as regulatory T cells (Tregs) and myeloid-derived suppressor cells, as well as upregulation of inhibitory receptors like TIM-3 and LAG-3, which reduce the efficacy of PD-1/PD-L1 blockade (19). TNBC often exhibits the characteristics of cold tumors, including a lack of tumor antigens, defects in antigen presentation, and insufficient T-cell infiltration in tumor tissues due to the failure of T cells to go home successfully. These mechanisms limit the effectiveness of ICIs (21). Moreover, MYC gene amplification is associated with the absence of immune cell infiltration, while mutations in the PI3K-AKT pathway may suppress the activation of innate immunity (22). To overcome these limitations, ongoing research explores combination therapies that include ICIs with chemotherapy, PARP inhibitors, and other immune-modulatory agents. These combinations aim to turn “cold” tumors into “hot” tumors, enhancing the immunogenicity of TNBC and improving patient outcomes (23, 24).

## Epigenetic mechanisms and cancer progression

Epigenetic modifications, including DNA methylation, histone modifications, and non-coding RNA (ncRNA) regulation, are pivotal in cancer development and progression. DNA methylation typically occurs at CpG islands in the promoter regions of genes, silencing tumor suppressor genes in many cancers (25). Histone modifications, such as acetylation and methylation, regulate chromatin structure, influencing gene expression. The enzymes involved in these modifications, such as histone deacetylases (HDACs) and histone methyltransferases, are often dysregulated in cancer, leading to aberrant transcriptional activation or repression (26). Non-coding RNAs, particularly microRNAs and long non-coding RNAs (lncRNAs), further modulate gene expression by affecting mRNA stability and translation (27). These epigenetic mechanisms are reversible, making them attractive targets for therapeutic intervention. Drugs targeting DNA methylation and histone modifications, such as DNA methyltransferase inhibitors (DNMTis) and HDAC inhibitors, are actively explored for cancer therapy, demonstrating promising results in hematologic and solid tumors (28).

**Figure 1:**
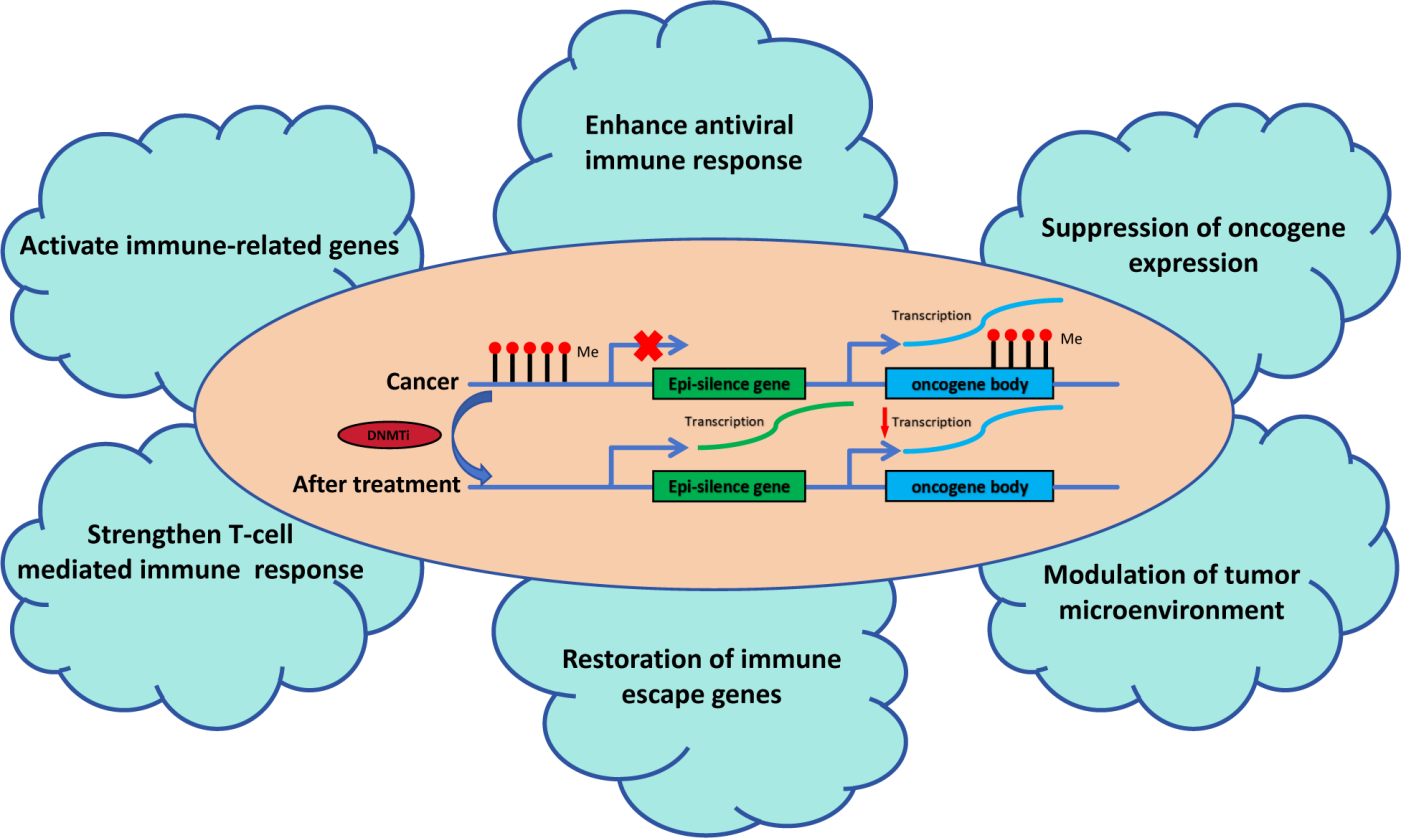
The role of DNA methyltransferase inhibitors in tumor immunity. DNMTis reactivate epi-silenced genes and suppress oncogene expression by reducing DNA methylation. This process enhances antiviral immune responses, activates immune-related genes, and strengthens T-cell-mediated immunity. It modulates the tumor microenvironment, restores immune escape genes, and suppresses oncogene-driven pathways, collectively improving tumor immune surveillance and therapeutic outcomes.

Epigenetic modifications, particularly DNA methylation, are intimately involved in TNBC’s immune evasion strategies. DNA methylation is a critical epigenetic mechanism that adds a methyl group to the cytosine ring within CpG islands, primarily in gene promoters. This process is catalyzed by DNA methyltransferases (DNMTs), such as DNMT1, DNMT3A, and DNMT3B, which play a crucial role in maintaining gene silencing (29, 30). Aberrant DNA methylation is prevalent in tumors, with variations in methylation levels across different regions exerting distinct impacts on gene transcription (**Figure 1**). Aberrant DNA methylation, particularly promoter hypermethylation, leads to the transcriptional repression of tumor suppressor genes, contributing to cancer initiation and progression (31). On the other hand, increased CpG site methylation within gene bodies can enhance gene expression, potentially by stabilizing the transcript (32). Blagitko-Dorfs (33) et al. discovered that the combined use of DNMT and HDAC inhibitors can downregulate oncogenes such as MYC in acute myeloid leukemia cells through the demethylation of gene bodies. This demonstrates that targeting gene body demethylation may represent a viable epigenetic therapeutic strategy. DNA methylation can silence the expression of immune-related genes, including those involved in antigen presentation and interferon signaling pathways, which are critical for an effective immune response (34).

## Interaction between DNA methylation and immune evasion

DNA demethylating agents have demonstrated therapeutic or diagnostic potential in hematological disorders (35), and in clinical trials involving patients with recurrent glioblastoma (36) and chemotherapy-resistant hepatocellular carcinoma (37). Furthermore, as reported by Linnekamp et al. (38), partial clinical responses have been observed in several solid tumors, including breast cancer, lung cancer, and colorectal cancer. These responses are characterized by gene demethylation and re-expression in certain patients. DNA methylation frequently silences immune-regulatory genes, impacting immune surveillance and evasion (39, 40). Hypermethylation leads to silencing tumor suppressor genes and immune-related genes, including those involved in antigen presentation and interferon signaling pathways (31). This silencing directly impacts immune escape mechanisms, as it reduces the expression of major histocompatibility complex (MHC) molecules and other immune-related markers, which are essential for effective immune surveillance and response (41). Hypermethylation of immune-regulatory genes like PD-L1 reduces the tumor’s ability to be recognized by cytotoxic T lymphocytes, promoting immune evasion and resistance to therapies (26). These findings suggest that targeting DNA methylation pathways could restore immune function and improve therapeutic outcomes in TNBC.

DNA methylation also plays a role in downregulating immune checkpoints, further enhancing immune evasion. The silencing of STING (stimulator of interferon genes), a crucial component of the innate immune response through DNA methylation, has been observed in TNBC. This silencing reduces the recruitment of immune cells to the tumor site, diminishing the anti-tumor immune response (42). Additionally, epigenetic modifications of immune-related genes contribute to the resistance in immunotherapy treatments, highlighting the potential of combining DNA methylation inhibitors with immunotherapy to overcome immune evasion (43).

## Epigenetic modifications and the tumor microenvironment

Epigenetic modifications also influence the tumor microenvironment, enhancing tumor survival and resistance to therapy. TNBC’s aggressive nature is partly due to the epigenetic remodeling of the extracellular matrix (ECM), which facilitates metastasis and therapeutic resistance (44). TNBC is notorious for its ability to evade the immune system by creating an immunosuppressive tumor microenvironment (TME) (45). One of the primary mechanisms involves the upregulation of immune checkpoint molecules such as PD-L1, which binds to PD-1 on T cells, effectively “turning off” the immune response and allowing the tumor to grow unchecked (46). This immune checkpoint blockade dampens cytotoxic T cell activity and enables TNBC cells to evade immune surveillance. TNBC tumors are also proficient at recruiting immunosuppressive cell populations, including regulatory T cells (Tregs) and myeloid-derived suppressor cells (MDSCs), further suppressing the anti-tumor immune response (47, 48).

Tregs (CD4^+^Foxp3^+^) cells, play a crucial role in immune evasion by secreting immunosuppressive cytokines such as IL-10 and TGF-β. These cytokines inhibit the activity of cytotoxic T cells and natural killer cells, thereby protecting the tumor from immune-mediated destruction (46). Similarly, MDSCs are known to suppress T cell proliferation and cytokine production, facilitating the tumor’s ability to escape immune detection (49). Together, these mechanisms create an immune-tolerant environment that allows TNBC to progress.

## Clinical relevance

DNA methylation biomarkers hold significant prognostic value in TNBC. Studies show that aberrant DNA methylation patterns are associated with poor prognosis and resistance to conventional therapies, such as chemotherapy and immunotherapy (50, 51). DNA methylation-induced silencing of tumor suppressor genes and immune-related genes contributes to chemotherapy resistance by preventing the reactivation of apoptotic pathways in cancer cells (52).

Moreover, DNA methylation has emerged as a mechanism of resistance to immunotherapies. Tumors with hypermethylated immune-regulatory genes often show reduced responsiveness to immune checkpoint inhibitors, which aim to enhance the body’s immune response against cancer (53). Combining DNMT inhibitors with immunotherapies has demonstrated the potential to reverse resistance and improve outcomes in TNBC patients.

## Combining DNA methylation with immunotherapy in TNBC

DNA methylation inhibitors, such as azacitidine and decitabine, are designed to reverse the epigenetic silencing of genes involved in immune regulation and tumor suppression. In the context of TNBC, these inhibitors can restore the expression of immune-related genes, enhancing the effectiveness of immunotherapies like ICIs (54). The hypermethylation of immune-related genes, particularly those involved in antigen presentation, limits immune cell infiltration and recognition, thus contributing to immune evasion in glioma (55). By reversing these epigenetic modifications, DNA methylation inhibitors promote immune activation, increasing tumor antigen visibility to cytotoxic T cells (56, 57). Preclinical studies have demonstrated the synergistic effects of combining DNA methylation inhibitors with ICIs in various cancers, including TNBC. For example, the combination of decitabine with anti-PD-1 therapy in mouse models of pancreatic cancer showed significantly improved survival rates, mediated by increased tumor-infiltrating lymphocytes and a reduction in immunosuppressive cell populations (58). Similar results have been observed in TNBC, where decitabine treatment increases the expression of antigen-presenting genes, such as MHC class I, leading to enhanced T cell-mediated immune responses (59). The underlying mechanisms of synergy between DNA methylation inhibitors and ICIs include enhanced immune cell infiltration, increased antigen presentation, and decreased levels of immunosuppressive factors within the TME. These effects are critical for converting immunologically “cold” tumors into “hot” tumors, which are more responsive to immune checkpoint blockade (53). Preclinical evidence suggests combining these therapies can induce durable anti-tumor responses, even in patients with advanced or resistant cancers.

## Challenges and limitations of combination therapy

One of the most significant challenges in treating TNBC with combination therapies, is the intrinsic heterogeneity of TNBC tumors. This diversity leads to varying responses to treatments, as different subclones within a tumor may respond differently to the same therapy (60). Recent studies suggest that identifying TNBC subtypes with specific DNA methylation or immune-related gene expression patterns may help personalize treatment strategies, improving the likelihood of therapeutic success (61). By classifying tumors into more homogeneous subgroups, therapies can be tailored to target the unique molecular features of each subtype, potentially overcoming the challenges posed by heterogeneity. Integrating large-scale multi-omics data requires addressing challenges such as computational demands, data standardization, and robust bioinformatics pipelines. From an ethical perspective, greater attention must be given to patient privacy, data security, and equitable access to personalized treatments derived from multi-omics data.

DNA methylation inhibitors face limitations in clinical application due to the broad spectrum of their target effects. Currently, these inhibitors are primarily used for refractory myelodysplastic syndromes and leukemia, where their therapeutic benefits have been clinically validated. However, the therapeutic efficacy remains unclear in solid tumors, as multiple genes may be epigenetically activated, complicating the treatment outcomes. DNA methylation inhibitors may also trigger compensatory or demethylation events that allow cancer cells to maintain their malignant phenotype despite treatment. Biomarkers such as PD-L1 expression, tumor mutational burden, and specific methylation patterns are being investigated to predict which patients will most likely benefit from these treatments (62).

The combination of DNA methylation inhibitors with immunotherapy introduces potential toxicity concerns, including immune-related adverse events (irAEs) and off-target effects. Immune checkpoint inhibitors can lead to autoimmune reactions, affecting organs such as the liver, lungs, and thyroid (38, 63). Similarly, DNA methylation inhibitors can cause off-target gene demethylation, potentially leading to unwanted gene expression changes that exacerbate toxicity. Studies have reported toxicities such as neutropenia, anemia, and elevated liver enzymes in patients receiving combination therapy.

## Future prospects

Biomarkers such as DNA methylation signatures, immune cell infiltration profiles, and circulating tumor DNA (ctDNA) are emerging as potential tools for predicting response to combination therapies in TNBC. Recent advances in single-cell sequencing have enabled more precise characterization of tumor heterogeneity and immune cell interactions, leading to the discovery of novel biomarkers that may guide treatment decisions (64). These biomarkers can also help monitor treatment response and detect early signs of resistance, allowing for timely adjustments in therapy (65). Developing predictive biomarkers such as tumor-infiltrating lymphocyte (TIL) profiles and PD-L1 expression could improve patient selection for immunotherapy-based treatments, increasing the likelihood of therapeutic success (66).

There is growing interest in exploring other epigenetic therapies, such as histone deacetylase (HDAC) inhibitors. HDAC inhibitors can modulate gene expression, enhancing immune recognition of tumors and sensitizing cancer cells to immune checkpoint inhibitors (67). Dual targeting of epigenetic mechanisms, such as combining HDAC inhibitors with DNA methylation or immune checkpoint inhibitors, may lead to more robust anti-tumor responses in TNBC (13). Preclinical studies have shown that HDAC inhibitors can enhance the infiltration of immune cells into tumors and promote the expression of tumor antigens, making them attractive candidates for combination therapies in TNBC (68).

## Conclusion

The combination of DNA methylation and immune checkpoint inhibitors holds promise for enhancing the therapeutic efficacy of TNBC. By reversing the epigenetic silencing of immune-regulatory genes, tumor visibility to immune cells can be improved, potentially overcoming the immune evasion characteristic of TNBC. While preclinical and early clinical trials have provided encouraging results, challenges such as tumor heterogeneity, resistance, and toxicity must be addressed. Future research should focus on utilizing multi-omics integration techniques within combination therapy groups to explore the mechanisms of personalized treatment, thereby defining specific therapeutic strategies to improve patient outcomes.

## Generative Al statement

The author(s) declare that Generative AI was used in the creation of this manuscript. During the preparation of this work, the authors employed ChatGPT-4.0 to enhance the language. The authors will subsequently conduct a comprehensive review and make necessary modifications, assuming full responsibility for the final published version.

## References

[1] Nedeljković M Damjanović A. Mechanisms of chemotherapy resistance in triple-negative breast cancer—How we can rise to the challenge. Cells. (2019) 8:957. doi: 10.3390/cells8090957 31443516 PMC6770896

[2] Gupta GK Collier AL Lee D Hoefer RA Zheleva V Siewertsz Van Reesema LL. Perspectives on triple-negative breast cancer: current treatment strategies, unmet needs, and potential targets for future therapies. Cancers. (2020) 12:2392. doi: 10.3390/cancers12092392 32846967 PMC7565566

[3] Patel R Kuwar U Dhote N Alexander A Nakhate K Jain P. Natural polymers as a carrier for the effective delivery of antineoplastic drugs. Curr Drug Delivery. (2023) 21:193–210. doi: 10.2174/1567201820666230112170035 36644864

[4] Dhote NS Patel RD Kuwar U Agrawal M Alexander A Jain P. Application of Thermoresponsive Smart Polymers based *in situ* Gel as a Novel Carrier for Tumor Targeting. Curr Cancer Drug Targets. (2023) 24:375–96. doi: 10.2174/1568009623666230803111718 37534485

[5] Raza MA Sharma MK Nagori K Jain P Ghosh V Gupta U. Recent trends on polycaprolactone as sustainable polymer-based drug delivery system in the treatment of cancer: Biomedical applications and nanomedicine. Int J Pharmaceutics. (2024) 666:124734. doi: 10.1016/j.ijpharm.2024.124734 39343332

[6] Sahu N Jain P Nagori K Ajazuddin. Recent advancement and novel treatment strategies for breast fibroadenoma: clinical approach and prospects. Curr Cancer Ther Rev. (2024) 20:1–11. doi: 10.2174/0115733947318171240802100419

[7] Gruosso T Gigoux M Manem VSK Bertos N Zuo D Perlitch I. Spatially distinct tumor immune microenvironments stratify triple-negative breast cancers. J Clin Invest. (2019) 129:1785–800. doi: 10.1172/JCI96313 PMC6436884 30753167

[8] Medina MA Oza G Sharma A Arriaga LG Hernández Hernández JM Rotello VM. Triple-negative breast cancer: A review of conventional and advanced therapeutic strategies. Int J Environ Res Public Health. (2020) 17:2078. doi: 10.3390/ijerph17062078 32245065 PMC7143295

[9] Shaffer C Robles A Risinger A Mooberry S. Abstract P2-13-01: Differential efficacies of DNA damaging agents in basal-like TNBC subtypes. Cancer Res. (2018) 78:P2–13–01. doi: 10.1158/1538-7445.SABCS17-P2-13-01

[10] Meisel J Douglass E Kalinsky K Griffiths LM Li Z Li X. Abstract P6-01-20: Luminal androgen receptor subtype and M2 macrophage signatures strongly associate with low pathological complete response rates and poor outcomes in patients with triple negative breast cancer receiving neoadjuvant chemotherapy. Cancer Res. (2023) 83:P6–01–20. doi: 10.1158/1538-7445.SABCS22-P6-01-20

[11] Abd El-Aziz YS Spillane AJ Jansson PJ Sahni S. Role of ABCB1 in mediating chemo-resistance of triple negative breast cancers. Bioscience Rep. (2021) 41(2):20204092. doi: 10.1042/BSR20204092 PMC7909869 33543229

[12] Ribeiro R Carvalho MJ Goncalves J Moreira JN. Immunotherapy in triple-negative breast cancer: Insights into tumor immune landscape and therapeutic opportunities. Front Mol Biosci. (2022) 9:903065. doi: 10.3389/fmolb.2022.903065 36060249 PMC9437219

[13] Lu Y Song Y Xu Y Ou N Liang Z Hu R. The prevalence and prognostic and clinicopathological value of PD-L1 and PD-L2 in renal cell carcinoma patients: a systematic review and meta-analysis involving 3,389 patients. Transl Androl Urol. (2020) 9:367–81. doi: 10.21037/tau.2020.01.21 PMC7215048 32420142

[14] Zhang H Dai Z Wu W Wang Z Zhang N Zhang L. Regulatory mechanisms of immune checkpoints PD-L1 and CTLA-4 in cancer. J Exp Clin Cancer Res. (2021) 40:367–81. doi: 10.1186/s13046-021-01987-7 PMC8178863 34088360

[15] Lu Y Kang J Luo Z Song Y Tian J Li Z. The prevalence and prognostic role of PD-L1 in upper tract urothelial carcinoma patients underwent radical nephroureterectomy: A systematic review and meta-analysis. Front Oncol. (2020) 10:1400. doi: 10.3389/fonc.2020.01400 32974145 PMC7472102

[16] Shiravand Y Khodadadi F Kashani SMA Hosseini-Fard SR Hosseini S Sadeghirad H. Immune checkpoint inhibitors in cancer therapy. Curr Oncol. (2022) 29:3044–60. doi: 10.3390/curroncol29050247 PMC9139602 35621637

[17] Eggermont AMM Maio M Robert C. Immune checkpoint inhibitors in melanoma provide the cornerstones for curative therapies. Semin Oncol. (2015) 42:429–35. doi: 10.1053/j.seminoncol.2015.02.010 25965361

[18] Willsmore ZN Coumbe BGT Crescioli S Reci S Gupta A Harris RJ. Combined anti-PD-1 and anti-CTLA-4 checkpoint blockade: Treatment of melanoma and immune mechanisms of action. Eur J Immunol. (2021) 51:544–56. doi: 10.1002/eji.202048747 33450785

[19] Zhang Y Chen H Mo H Hu X Gao R Zhao Y. Single-cell analyses reveal key immune cell subsets associated with response to PD-L1 blockade in triple-negative breast cancer. Cancer Cell. (2021) 39:1578–93.e8. doi: 10.1016/j.ccell.2021.09.010 34653365

[20] Saleh R Toor SM Khalaf S Elkord E. Breast cancer cells and PD-1/PD-L1 blockade upregulate the expression of PD-1, CTLA-4, TIM-3 and LAG-3 immune checkpoints in CD4+ T cells. Vaccines. (2019) 7. doi: 10.3390/vaccines7040149 PMC6963740 31614877

[21] Bonaventura P Shekarian T Alcazer V Valladeau-Guilemond J Valsesia-Wittmann S Amigorena S. Cold tumors: A therapeutic challenge for immunotherapy. Front Immunol. (2019) 10:168. doi: 10.3389/fimmu.2019.00168 30800125 PMC6376112

[22] Xiao Y Ma D Zhao S Suo C Shi J Xue zhu M. Multi-omics profiling reveals distinct microenvironment characterization and suggests immune escape mechanisms of triple-negative breast cancer. Clin Cancer Res. (2019) 25:5002–14. doi: 10.1158/1078-0432.CCR-18-3524 30837276

[23] Telli ML Stover DG Loi S Aparicio S Carey LA Domchek SM. Homologous recombination deficiency and host anti-tumor immunity in triple-negative breast cancer. Breast Cancer Res Treat. (2018) 171:21–31. doi: 10.1007/s10549-018-4807-x 29736741

[24] Keenan TE Tolaney SM. Role of immunotherapy in triple-negative breast cancer. J Natl Compr Cancer Network. (2020) 18:479–89. doi: 10.6004/jnccn.2020.7554 32259782

[25] Jin N George TL Otterson GA Verschraegen C Wen H Carbone D. Advances in epigenetic therapeutics with focus on solid tumors. Clin Epigenet. (2021) 13:83. doi: 10.1186/s13148-021-01069-7 PMC8056722 33879235

[26] Li Y Li Z Zhu WG. Molecular mechanisms of epigenetic regulators as activatable targets in cancer theranostics. Curr Medicinal Chem. (2019) 26:1328–50. doi: 10.2174/0929867324666170921101947 28933282

[27] Gray JS Wani SA Campbell MJ. Epigenomic alterations in cancer: mechanisms and therapeutic potential. Clin Sci. (2022) 136:473–92. doi: 10.1042/CS20210449 35383835

[28] Patnaik S Anupriya. Drugs targeting epigenetic modifications and plausible therapeutic strategies against colorectal cancer. Front Pharmacol. (2019) 10:588. doi: 10.3389/fphar.2019.00588 31244652 PMC6563763

[29] Stresemann C Lyko F. Modes of action of the DNA methyltransferase inhibitors azacytidine and decitabine. Int J Cancer. (2008) 123:8–13. doi: 10.1002/ijc.v123:1 18425818

[30] Chen S Jiang Y Wang C Tong S He Y Lu W. Epigenetic clocks and gliomas: unveiling the molecular interactions between aging and tumor development. Front Mol Biosci. (2024) 11:1446428. doi: 10.3389/fmolb.2024.1446428 39130373 PMC11310061

[31] Yu J Zayas J Qin B Wang L. Targeting DNA methylation for treating triple-negative breast cancer. Pharmacogenomics. (2019) 20:1151–7. doi: 10.2217/pgs-2019-0078 PMC7026764 31755366

[32] Wang Q Xiong F Wu G Liu W Chen J Wang B. Gene body methylation in cancer: molecular mechanisms and clinical applications. Clin Epigenet. (2022) 14:154. doi: 10.1186/s13148-022-01382-9 PMC9706891 36443876

[33] Blagitko-Dorfs N Schlosser P Greve G Pfeifer D Meier R Baude A. Combination treatment of acute myeloid leukemia cells with DNMT and HDAC inhibitors: predominant synergistic gene downregulation associated with gene body demethylation. Leukemia. (2019) 33:945–56. doi: 10.1038/s41375-018-0293-8 30470836

[34] Shadbad MA Safaei S Brunetti O Derakhshani A Lotfinejad P Mokhtarzadeh A. A systematic review on the therapeutic potentiality of PD-L1-inhibiting microRNAs for triple-negative breast cancer: toward single-cell sequencing-guided biomimetic delivery. Genes. (2021) 12:1206. doi: 10.3390/genes12081206 34440380 PMC8391239

[35] Agrawal K Das V Vyas P Hajdúch M. Nucleosidic DNA demethylating epigenetic drugs – A comprehensive review from discovery to clinic. Pharmacol Ther. (2018) 188:45–79. doi: 10.1016/j.pharmthera.2018.02.006 29454856

[36] Kirkin AF Dzhandzhugazyan KN Guldberg P Fang JJ Andersen RS Dahl C. Adoptive cancer immunotherapy using DNA-demethylated T helper cells as antigen-presenting cells. Nat Commun. (2018) 9:785. doi: 10.1038/s41467-018-03217-9 29511178 PMC5840134

[37] Galle E Thienpont B Cappuyns S Venken T Busschaert P Van Haele M. DNA methylation-driven EMT is a common mechanism of resistance to various therapeutic agents in cancer. Clin Epigenet. (2020) 12:27. doi: 10.1186/s13148-020-0821-z PMC7023776 32059745

[38] Linnekamp JF Butter R Spijker R Medema JP Van Laarhoven HWM. Clinical and biological effects of demethylating agents on solid tumours – A systematic review. Cancer Treat Rev. (2017) 54:10–23. doi: 10.1016/j.ctrv.2017.01.004 28189913

[39] Casalino L Verde P. Multifaceted roles of DNA methylation in neoplastic transformation, from tumor suppressors to EMT and metastasis. Genes. (2020) 11:922. doi: 10.3390/genes11080922 32806509 PMC7463745

[40] Chen C Wang Z Ding Y Wang L Wang S Wang H. DNA methylation: from cancer biology to clinical perspectives. Front Bioscience. (2022) 27:326. doi: 10.31083/j.fbl2712326 36624943

[41] Liu P Yang F Zhang L Hu Y Chen B Wang J. Emerging role of different DNA methyltransferases in the pathogenesis of cancer. Front Pharmacol. (2022) 13:958146. doi: 10.3389/fphar.2022.958146 36091786 PMC9453300

[42] Lee K Lin CC Servetto A Bae J Kandagatla V Ye D. Epigenetic repression of STING by MYC promotes immune evasion and resistance to immune checkpoint inhibitors in triple negative breast cancer. Cancer Immunol Res. (2022) 10:829–43. doi: 10.1158/2326-6066.CIR-21-0826 PMC9250627 35561311

[43] Gomez S Tabernacki T Kobyra J Roberts PA Chiappinelli KB. Combining epigenetic and immune therapy to overcome cancer resistance. Semin Cancer Biol. (2019) 65:99–113. doi: 10.1016/j.semcancer.2019.12.019 PMC7308208 31877341

[44] Zolota V Tzelepi V Piperigkou Z Kourea H Papakonstantinou E Argentou MI. Epigenetic alterations in triple-negative breast cancer—The critical role of extracellular matrix. Cancers. (2021) 13:713. doi: 10.3390/cancers13040713 33572395 PMC7916242

[45] Feng D Zhang F Li D Shi X Xiong Q Wei Q. Developing an immune-related gene prognostic index associated with progression and providing new insights into the tumor immune microenvironment of prostate cancer. Immunology. (2022) 166:197–209. doi: 10.1111/imm.v166.2 35271752

[46] Zhulai G Oleinik E. Targeting regulatory T cells in anti-PD-1/PD-L1 cancer immunotherapy. Scandinavian J Immunol. (2021) 95:e13129. doi: 10.1111/sji.13129 34936125

[47] Kajihara N Kobayashi T Otsuka R Nio-Kobayashi J Oshino T Takahashi M. Tumor-derived interleukin-34 creates an immunosuppressive and chemoresistant tumor microenvironment by modulating myeloid-derived suppressor cells in triple-negative breast cancer. Cancer Immunol Immunother. (2023) 72:851–64. doi: 10.1007/s00262-022-03293-3 PMC10992550 36104597

[48] Tao L Cheng G Lv F Wang R Yang N Xing Z. Synergistic strategy based on mild phototherapy and deep tumor hypoxia reversal comprehensively remodels the tumor microenvironment for improved immunotherapy. Chem Eng J. (2023) 472:145092. doi: 10.1016/j.cej.2023.145092

[49] Takeyama Y Kato M Tamada S Azuma Y Shimizu Y Iguchi T. Myeloid-derived suppressor cells are essential partners for immune checkpoint inhibitors in the treatment of cisplatin-resistant bladder cancer. Cancer Lett. (2020) 479:89–99. doi: 10.1016/j.canlet.2020.03.013 32200039

[50] Lin F Huang J Zhu W Jiang T Guo J Xia W. Prognostic value and immune landscapes of TERT promoter methylation in triple negative breast cancer. Front Immunol. (2023) 14:1218987. doi: 10.3389/fimmu.2023.1218987 37575241 PMC10416624

[51] Gómez-Miragaya J Morán S Calleja-Cervantes ME Collado-Sole A Paré L Gómez A. The altered transcriptome and DNA methylation profiles of docetaxel resistance in breast cancer PDX models. Mol Cancer Res. (2019) 17:2063–76. doi: 10.1158/1541-7786.MCR-19-0040 PMC7616969 31320385

[52] Zorzan E Elgendy R Guerra G Da Ros S Gelain ME Bonsembiante F. Hypermethylation-mediated silencing of CIDEA, MAL and PCDH17 tumour suppressor genes in canine DLBCL: from multi-omics analyses to mechanistic studies. Int J Mol Sci. (2022) 23:4021. doi: 10.3390/ijms23074021 35409379 PMC9000013

[53] Hu C Liu X Zeng Y Liu J Wu F. DNA methyltransferase inhibitors combination therapy for the treatment of solid tumor: mechanism and clinical application. Clin Epigenet. (2021) 13:166. doi: 10.1186/s13148-021-01154-x PMC8394595 34452630

[54] Wong KF. DNMT1: A key drug target in triple-negative breast cancer. Seminars in Cancer Biology. (2021) 72:198–213. doi: 10.1016/j.semcancer.2020.05.010 32461152

[55] McClellan BL Haase S Nunez FJ Alghamri MS Dabaja AA Lowenstein PR. Impact of epigenetic reprogramming on antitumor immune responses in glioma. J Clin Invest. (2023) 133(2).e163450. doi: 10.1172/JCI163450 36647827 PMC9843056

[56] Giri A Aittokallio T. DNMT inhibitors increase methylation in the cancer genome. Front Pharmacol. (2019) 10:385. doi: 10.3389/fphar.2019.00385 31068808 PMC6491738

[57] Katarzyna R Lucyna B. Epigenetic therapies in patients with solid tumors: Focus on monotherapy with deoxyribonucleic acid methyltransferase inhibitors and histone deacetylase inhibitors. J Can Res Ther. (2019) 15(5):961. doi: 10.4103/jcrt.JCRT_403_17 31603095

[58] Gonda T Fang J Salas M Do C Hsu E Zhukovskaya A. A DNA hypomethylating drug alters the tumor microenvironment and improves the effectiveness of immune checkpoint inhibitors in a mouse model of pancreatic cancer. Cancer Res. (2020) 80:4754–67. doi: 10.1158/0008-5472.CAN-20-0285 PMC9296074 32816859

[59] Alamoudi M Chipman M Deleso-Frechette F Liu E Zhang R Wang Z. A therapeutic strategy to inhibit Wnt signaling also reprograms breast tumor-immune cell interactions: Perspectives for conferring immune checkpoint inhibitor susceptibility. Cancer Immunol Res. (2020) 8:A39–9. doi: 10.1158/2326-6074.TUMIMM19-A39

[60] Ge JY Shu S Kwon M Jovanović B Murphy K Gulvady A. Acquired resistance to combined BET and CDK4/6 inhibition in triple-negative breast cancer. Nat Commun. (2020) 11:2350. doi: 10.1038/s41467-020-16170-3 32393766 PMC7214447

[61] Pack C Bommireddy R Munoz LE Patel JM Bozeman E Dey P. Tumor membrane-based vaccine immunotherapy in combination with anti-CTLA-4 antibody confers protection against immune checkpoint resistant murine triple-negative breast cancer. Hum Vaccines Immunotherapeutics. (2020) 16:3184–93. doi: 10.1080/21645515.2020.1754691 PMC8641616 32530786

[62] Raz D. Targeting epigenetic regulators in cancer to overcome targeted therapy resistance. Targeted Therapies Lung Cancer. (2019), 217–32. doi: 10.1007/978-3-030-17832-1_11

[63] Damodaran S Liu D Schwartz J Valero V Ramirez D Saleem S. A phase Ib trial of bintrafusp alfa and eribulin in patients with metastatic triple-negative breast cancer (TNBC). Cancer Res. (2023) 83:P3–02–03. doi: 10.1158/1538-7445.sabcs22-p3-02-03

[64] Yu JX Guo Z Wang L. Progress and challenges of immunotherapy predictive biomarkers for triple negative breast cancer in the era of single-cell multi-omics. Life. (2023) 13:1189. doi: 10.3390/life13051189 37240834 PMC10221869

[65] Llinàs-Arias P Íñiguez-Muñoz S McCann K Voorwerk L Orozco JIJ Ensenyat-Mendez M. Epigenetic regulation of immunotherapy response in triple-negative breast cancer. Cancers. (2021) 13:4139. doi: 10.3390/cancers13164139 34439290 PMC8394958

[66] Shen H Yang ESH Conry M Fiveash J Contreras C Bonner JA. Predictive biomarkers for immune checkpoint blockade and opportunities for combination therapies. Genes Dis. (2019) 6:232–46. doi: 10.1016/j.gendis.2019.06.006 PMC6997608 32042863

[67] Borcoman E Kamal M Marret G Dupain C Castel-Ajgal Z Le Tourneau C. HDAC inhibition to prime immune checkpoint inhibitors. Cancers. (2021) 14:66. doi: 10.3390/cancers14010066 35008230 PMC8750966

[68] Messerli S Hoffman MM Zylla JS Gnimpieba E Miskimins KW. Examination of therapeutic potential of epigenetic modulation by dual HDAC-LSD1 inhibition in murine models of triple negative breast cancer (TNBC). Cancer Res. (2020) 80:1761. doi: 10.1158/1538-7445.AM2020-1761

